# The golden retriever model of Duchenne muscular dystrophy

**DOI:** 10.1186/s13395-017-0124-z

**Published:** 2017-05-19

**Authors:** Joe N. Kornegay

**Affiliations:** Department of Veterinary Integrative Biosciences, College of Veterinary Medicine and Biomedical Sciences, Texas A&M University, Mail Stop 4458, College Station, TX 77843-4458 USA

**Keywords:** Duchenne muscular dystrophy (DMD), Golden retriever muscular dystrophy (GRMD), Animal models, Preclinical studies

## Abstract

Duchenne muscular dystrophy (DMD) is an X-linked disease caused by mutations in the *DMD* gene and loss of the protein dystrophin. The absence of dystrophin leads to myofiber membrane fragility and necrosis, with eventual muscle atrophy and contractures. Affected boys typically die in their second or third decade due to either respiratory failure or cardiomyopathy. Despite extensive attempts to develop definitive therapies for DMD, the standard of care remains prednisone, which has only palliative benefits. Animal models, mainly the mdx mouse and golden retriever muscular dystrophy (GRMD) dog, have played a key role in studies of DMD pathogenesis and treatment development. Because the GRMD clinical syndrome is more severe than in mice, better aligning with the progressive course of DMD, canine studies may translate better to humans. The original founder dog for all GRMD colonies worldwide was identified in the early 1980s before the discovery of the *DMD* gene and dystrophin. Accordingly, analogies to DMD were initially drawn based on similar clinical features, ranging from the X-linked pattern of inheritance to overlapping histopathologic lesions. Confirmation of genetic homology between DMD and GRMD came with identification of the underlying GRMD mutation, a single nucleotide change that leads to exon skipping and an out-of-frame *DMD* transcript. GRMD colonies have subsequently been established to conduct pathogenetic and preclinical treatment studies. Simultaneous with the onset of GRMD treatment trials, phenotypic biomarkers were developed, allowing definitive characterization of treatment effect. Importantly, GRMD studies have not always substantiated findings from mdx mice and have sometimes identified serious treatment side effects. While the GRMD model may be more clinically relevant than the mdx mouse, usage has been limited by practical considerations related to expense and the number of dogs available. This further complicates ongoing broader concerns about the poor rate of translation of animal model preclinical studies to humans with analogous diseases. Accordingly, in performing GRMD trials, special attention must be paid to experimental design to align with the approach used in DMD clinical trials. This review provides context for the GRMD model, beginning with its original description and extending to its use in preclinical trials.

## Background

Duchenne muscular dystrophy (DMD) is a devastating X-linked inherited degenerative muscle disease [1] affecting ~1 in 4000–6000 boys [2]. Mutations in the *DMD* gene limit production of the protein, dystrophin, resulting in loss of myofiber membrane integrity and repeated cycles of necrosis and regeneration [1]. Muscle is gradually replaced with fibrous connective tissue and fat, leading to weakness and debilitating contractures. Eventual involvement of respiratory muscles and the heart causes cardiopulmonary failure and death in the second to third decade of life. Although the molecular basis for DMD was defined 30 years ago, glucocorticoids and supportive therapy remain the standard of care.

Prior to the discovery of the *DMD* gene and dystrophin protein in the 1980s, there were no definitive genetic animal models for DMD. Various inherited and experimental primary myopathies in animals, most notably in mice, chickens, and hamsters, were studied in an effort to gain insight into the pathogenesis and potential treatment of the human dystrophies [3]. The most obvious discrepancy in these models related to their autosomal versus X-linked pattern of inheritance. While these animal studies provided useful insight on disease pathogenesis, their overall value was questioned [4].

Subsequently, spontaneous genetically homologous dystrophinopathies have been identified in several mammalian species, including mice and dogs. Because the phenotype of dystrophic dogs more closely mirrors that of DMD, pathogenetic and preclinical treatment studies may better translate to humans. Most canine studies have been conducted in the golden retriever muscular dystrophy (GRMD) model, which occurs due to a spontaneous splice site mutation in the *DMD* gene. In this review, fundamental early observations that hinted at the membranal nature of both DMD and GRMD are covered first, followed by a discussion of molecular studies that identified the *DMD* gene and dystrophin protein. Challenges facing physicians and scientists in translating therapies from animals to humans are then discussed, with emphasis on the importance of first and foremost establishing safety. The review concludes with an overview of the role of animal models and, in particular, GRMD in treatment development.

## Disease pathogenesis: the membrane theory

**Fig. 1:**
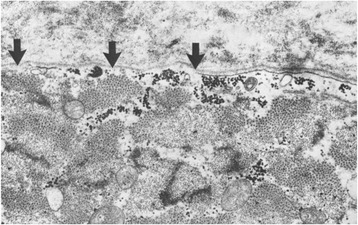
Characteristic myofiber membrane lesion in DMD. Electron photomicrograph demonstrates lack of continuity of the myofiber (sarcolemmal) membrane (*arrows*), while the basal lamina remains intact. The myofiber architecture is disrupted subjacent to the membrane lesion. Original magnification 26,000. From reference [8]

Well before the molecular age allowed identification of disease-causing genes, physicians and scientists relied on clinical clues and their intuition to infer disease pathogenesis. Much early attention focused on the so-called membrane theory of DMD, as stated by Rowland, “The functional genetic fault of DMD affects an enzyme or structural protein which is decreased in amount or rendered functionally abnormal because of an altered amino acid sequence. In either case, the altered protein results in abnormal composition and altered function of muscle cell surface membranes” [4]. The membrane theory originated with the observation that enzymes, such as aldolase and phosphorylase, were decreased in muscle [5] and elevated in serum [6, 7]. This was presumed to occur because of damage to the myofiber membrane, the sarcolemma. In fact, elevations of creatine phosphokinase (CPK), now typically shortened to creatine kinase (CK), had become particularly useful in the diagnosis of DMD [6]. Additional support for the membrane theory came from ultrastructural studies showing defects in the sarcolemma (Fig. 1) that purportedly allowed enzyme leakage [8–11]. Concomitant influx of calcium was hypothesized to lead to fiber hypercontraction or protease activation, each of which could contribute to the characteristic myofiber (hyaline) necrosis seen in DMD. In support of this mechanistic disease association, the membrane lesions sometimes overlay wedge-shaped areas of focal necrosis, so called delta lesions [10] (see Fig. 4).

**Fig. 2:**
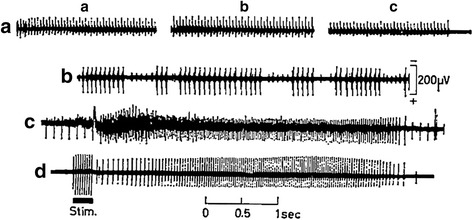
Spontaneous EMG activity in muscle disease. Activity, termed pseudomyotonic bursts, that begins and ends abruptly, in the hypertrophied calf muscle of a DMD patient (**a**, **b**), contrasts with waxing and waning myotonic activity from a forearm extensor of a myotonic dystrophy patient (**c**, **d**). The activity in **a** was recorded from two fibers discharging at 22 and 11/s over a 45-s period with the three bursts (*a*–*c*) separated by 20 s. Bursts in **b** had a frequency of 15/s. Increasing and decreasing activity in **c** (maximum 50/s) and **d** (maximum 35/s) were induced by movement of the concentric recording electrode and direct stimuli, respectively. From reference [12]

In addition to enzyme changes and sarcolemma lesions, electromyography (EMG) provided a third less discussed marker of potential membrane involvement. As with other myopathies, DMD is characterized by low amplitude, short-duration motor unit potentials, with associated spontaneous activity ranging from fibrillation potentials to complex repetitive discharges (CRDs) [12–15]. The occurrence of CRDs is of particular interest, given their predominance on EMG studies in canine dystrophinopathies (see below). Previously called pseudomyotonia or bizarre high-frequency discharges, CRDs are high frequency spontaneous potentials that begin and end abruptly [16, 17] (Fig. 2). This pattern contrasts with true myotonic discharges that wax and wane, creating a characteristic “dive bomber” sound [17]. While spontaneous activity on EMG may occur with either neuropathies or myopathies, CRDs occur preferentially in myopathies [18] and point to myofiber membrane involvement [19]. Buchtral and Rosenfalck reported *pseudomyotonic bursts* in human progressive muscular dystrophy patients in their 1963 monograph [12] (Fig. 2). Others have shown that CRDs occur more often than additional forms of pathologic spontaneous activity in DMD versus the less clinically severe dystrophinopathy, Becker muscular dystrophy (BMD) (see below), and even more so, when compared to the other dystrophies [14, 15]. In general, CRDs are thought to occur through ephaptic transmission of action potentials between muscle fibers, although the involved mechanisms are not well understood [17, 20]. In principle, membrane lesions that limit chloride or enhance sodium conductance into the myofiber could shift the resting membrane potential towards the threshold for depolarization, with associated repetitive electrical discharges [21]. Consistent with this observation, mutations in chloride (CLCN1) and sodium (SCN4A) skeletal muscle channel genes have been incriminated in the myotonias [16]. Moreover, dysregulation of Na_V_1.4, the protein product of SCN4A, leads to increased intracellular sodium and cell death in mdx mice [22], which also express CRDs [23, 24].

## The *DMD* gene, dystrophin protein, and dystrophin-glycoprotein complex

The mode of inheritance for DMD was long suspected to be X-linked because of the disease’s predominance in males. While an X-linked pattern was ultimately corroborated by pedigree studies [25, 26], this interpretation was clouded by the rare occurrence of a similar clinical syndrome in girls [27]. Some such cases were thought to occur due to disproportionate inactivation of the normal X chromosome in female DMD carriers, as was subsequently well documented [28]. Others were associated with autosomal translocations within the Xp21 region of the short arm of the X-chromosome, pointing to this area as a likely site of the *DMD* gene [29]. This putative site was confirmed when restriction fragment length polymorphisms (RFLPs; markers) cleaved from DNA were shown to flank the Xp21 locus [30]. An analogous association was made with the less severe clinical syndrome of BMD [31]. Following closely on this work, Kunkel et al. capitalized on a DMD patient with a cytogenetically visible Xp21 deletion that also included genes for three other diseases [32, 33]. Several DNA fragments absent from the patient were cloned to provide linkage markers for the *DMD* locus. In a follow-up multicenter study, DNA from over a thousand DMD and BMD patients was probed using these clones, with 6.5% showing deletions at the DXS21 locus [34]. Using the DXS21 locus as the starting point, chromosomal walking was employed to map and clone the entire 14 kb *DMD* gene transcript [35]. Dystrophin, the 425 kd protein product of the *DMD* gene, was characterized and found to be absent in both DMD patients and the mdx mouse [36]. Tying matters together further, the molecular basis for DMD and BMD was shown to depend on whether the underlying mutation maintained (BMD; in frame) or disrupted (DMD; out of frame) the three nucleotide (codon) reading frame for amino acids [37]. Subsequent studies have established that deletions (60%) and duplications (5%) account for ~65% of DMD and BMD mutations and that they tend to concentrate in two so-called hot spot areas near the N-terminus (exons 3-7) and within the central rod domain (exons 45-53) [38–41]. Definition of the specific extent and location of the mutation has become critical with the advent of antisense oligonucleotide therapies intended to reestablish the reading frame (see below in the context of canine *DMD* gene mutations) [42]. In tandem with early studies of the *DMD* gene, Campbell and Kahl published work that led to a series of comprehensive studies linking dystrophin with additional proteins and sugar moieties to form the dystrophin-glycoprotein complex [43]. A number of other muscular dystrophies subsequently have been causally associated with mutations in genes coding for structural proteins and glycosylating enzymes within this complex [44, 45].

## The search for a cure

The approach to DMD treatment can be divided into two periods. The first predated identification of the *DMD* gene and dystrophin protein when management was largely symptomatic or employed drugs directed at disease mechanisms inferred from pathologic changes. Extending from this period, the second phase has focused on directed genetic and cellular approaches that offer the potential for cure. Soon after the discovery of the *DMD* gene and dystrophin protein, the “*Journal of Child Neurology*” published three perspectives from Jan Witkowski [46], Victor Dubowitz [47], and Jerry Lewis [48], the first two commenting as a scientist and clinician and Mr. Lewis as a longstanding champion for the Muscular Dystrophy Association and “Jerry’s Kids.” Key comments from each of their commentaries are reproduced here:

This new knowledge has tremendous implications for the physician caring for DMD patients and their families, and it has led to new methods for diagnosis, prognosis, and genetic counseling. In the not-too-distant future, this research may lead to therapies for DMD. Jan Witkowski

Although it may be exciting to speculate on the possibility of gene therapy in muscular dystrophy, it seems extremely unlikely that this could become a reality in the foreseeable future. Victor Dubowitz

I don’t pretend to understand the language of science or comprehend the many complex research findings discussed in these articles. But there’s a basic, underlying message I do understand – a message of excitement that we’ve scored long-awaited victories against a disease that cripples and kills thousands of children, and of hope that these victories will soon lead us to a cure or a treatment. Jerry Lewis

Reflecting on these comments in hindsight garnered over 30 years, each appears to have gotten things just about right. As Dr. Witkowski predicted, the discovery of the *DMD* gene and dystrophin protein has advanced the fields of diagnosis and genetic counseling and set the stage for more directed DMD treatments, to include gene and cell-based approaches. And, who could argue with Jerry Lewis, who pointed to the tremendous energy that these discoveries unleashed. But, Dr. Dubowitz’s cautionary note was also prophetic. Progress on genetic therapies has been frustratingly slow, with a number of approaches failing or being delayed by unforeseen complications. The fact that glucocorticoids remain the standard of care for DMD provides stark evidence of the challenges of implementing genetic and cellular therapies [49, 50].

## The long and winding road to drug approval

The road to drug approval is often difficult, with less than 20% of Phase II trials being successful, typically due to either a lack of efficacy or safety concerns [51, 52]. Failure to demonstrate efficacy is caused, in part, by challenges in accruing sufficient patients to achieve necessary power [53]. This issue is exaggerated with rare (orphaned) diseases such as DMD. The Orphan Drug Act of 1983 provided various Food and Drug Administration (FDA) incentives, including expedited review and delayed approval of competing drugs, to facilitate development of therapeutics [54]. Motivated by these incentives, there has been a marked uptick in approaches directed at Duchenne patients. A total of 206 studies of DMD diagnostic tests and treatment trials in various stages of initial enrollment to termination are currently listed on the NIH Clinical Trials.gov website (https://clinicaltrials.gov/ct2/results?term=Duchenne+Muscular+Dystrophy&pg=2). Results of these new treatments have been mixed [49]. On a promising note, ataluren, the first drug approved to specifically target the underlying *DMD* gene mutation, showed potential benefit in an initial phase 2b study [55, 56] and patients are now being enrolled in a larger phase 3-trial (https://clinicaltrials.gov/ct2/show/NCT02090959?term=ataluren&rank=1). However, other recent clinical trials, including the failure of phophodiester-5 inhibitors to translate to Duchenne and Becker patients [57, 58] and ongoing questions surrounding efficacy of the exon-skipping strategies [59, 60], have illustrated the complexities surrounding treatment development.

## Doctor, do no harm

In treating patients, physicians have been guided by a set of ethical principles that date to ancient Greece and are embodied in the Hippocratic oath. Indeed, some form of this oath is taken by newly graduating physicians and should guide all who practice clinical medicine. While not specifically included in the original oath, newer versions typically include language to the effect of, “first, do no harm.” Safety also lies at the heart of the FDA's approval process for an investigational new drug (IND), as detailed in FDA Code of Federal Regulations (CFR) Title 21, Part 312 (accessdata.fda.gov/scripts/cdrh/cfdocs/cfcfr/CFRSearch.cfm?CFRPart = 312), beginning with preclinical testing in animals and the subsequent phased process for human clinical trials. Smaller populations of human volunteers are first evaluated to establish safety (Phase 1) before larger clinical groups are tested to determine efficacy (Phases 2-4).

Preclinical animal testing has played a critical role in demonstrating drug safety, extending from FDA policies formulated over the past 50 years to the IND protocol used today. Acute, subacute, and chronic dosing studies are required in a rodent and second (often dog) species [61, 62]. For sake of pediatric populations, emphasis has been placed on the use of analogous juvenile animals to better predict potential developmental side effects (see “GRMD in preclinical trials: experimental design” section below) [63]. The FDA has also recently released rigorous guidelines for gene and cell-based therapies [64, 65]. Germane to this review, these guidelines emphasize choosing animal models likely to demonstrate a biological response, such as immunity, that would be expected in humans. Moreover, disease models were recommended over normal animals because of their greater likelihood to predict the risk-benefit ratio of the investigational product.

## The golden retriever model

### Animal models: an overview

Safety considerations discussed above point to the importance of careful design of preclinical studies, to include animal model selection. In this regard, a 1985 National Research Council report emphasized that biological modeling could be by either *analogy* or *homology* [66]. Analogy implies a point-by-point relationship between one structure or process to another, while homology suggests a shared evolutionary history and matching DNA makeup. Before the discovery of the *DMD* gene in 1987, it was not possible to identify true genetically homologous animal models. Investigators had to rely on models that were neither analogous phenotypically nor genetically homologous [3]. Naturally occurring genetic DMD mammalian models have subsequently been defined in mice [67–69], dogs [70], cats [71, 72], and pigs [73]. As would be expected, given inherent biologic differences between quadruped animals and humans, none of these mammalian DMD models are fully analogous clinically. Most notably, mdx mice are mildly affected, with near-normal life expectancy. Dystrophic cats have a curious hypertrophic myopathy and are prone to a malignant-hyperthermia like syndrome [74]. Dogs have a severe phenotype that more closely mirrors that of DMD. But, as discussed below, dystrophic dogs may stabilize after ~6 months of age and often live well into adulthood [75]. Although knockout and spontaneous dystrophic pigs have not yet been fully characterized, one knock out model had an unexpectedly severe phenotype (Rogers C, personal communication, 2014). The lack of analogy between the phenotypes of different animal models and DMD inherently limits conclusions that can be reached from mechanistic and preclinical studies. A rat model, created by molecularly targeting *DMD* gene exon 23, could have advantages over the thus-far described mdx and larger spontaneous mammalian models [76].

Potential DMD treatments have generally been tested initially in the mdx mouse, often establishing proof of concept for the particular approach [77]. Major advantages of the mdx mouse include its consistent phenotype and relatively modest expense, which allows multiple variables to be tested through reasonably powered studies. On the other hand, the mouse’s small size limits assessment of scalable variables such as cell migration or drug diffusion [78]. Perhaps even more importantly, mdx mouse preclinical trials have generally not identified treatment complications. This has been particularly problematic with their failure to predict immunologic side effects of gene and cell therapies. These advantages and limitations are essentially reversed for dystrophic dogs. Expense of maintaining dogs limits the number of variables that can be tested and phenotypic variation further reduces the power that can be achieved. With that said, the dog’s larger size and outbred nature allows for better modeling of scalable variables such as cell diffusion and the immune response to biologics. A general paradigm has evolved, whereby initial testing is done in mdx mice and, pending positive results, follow-up studies in dystrophic dogs are considered.

### Animal models: the two cultures of drug discovery

Considerable attention has recently been focused on the failure of preclinical treatment trials to translate to human patients. Various reasons have been offered to account for this disconnect. A key factor relates to what has been termed the “two cultures phenomenon” [79]. Put simply, preclinical studies are often as loose as clinical trials are rigorous. Too little attention is paid to basic tenets of experimental design, extending from power analysis of the biomarker used to assess efficacy to the need for blinding [80, 81]. This problem extends to the level of detail provided on experimental design in grant applications and also to the degree to which it is considered in the review process. Understandably, the review process places considerable emphasis on a proposal’s innovative approach and potential impact. Unfortunately, less attention is sometimes paid to experimental design. Canine and other large animal models are particularly vulnerable in this area. Legitimate animal availability and budgetary issues consistently preclude conduct of a *perfect study* that is sufficiently powered and allows all variables to be considered. Accordingly, large animal studies must be focused and resist the temptation to explore a tangential issue.

### Animal models: a change in mindset

As detailed above, preclinical animal studies help to inform the drug discovery process for both efficacy and safety. Safety is well represented in classical toxicologic assays that demonstrate potential off-target effects of a drug, as with hepatotoxicy. However, safety has often taken a backseat to efficacy in preclinical treatment trials. Investigators seem overly motivated to show that a treatment “works” versus pointing out that it failed or had deleterious consequences. Results that identify limitations of a treatment are said to be “disappointing” and, potentially, not even deserving of publication [81]. One could argue that this is precisely the wrong attitude. The greatest shortcoming of an animal model is not that it failed to demonstrate efficacy but that it failed to identify a potential risk. That it did not identify a worst-case scenario that led to a set of serious side effects. Stated another way, an animal model has failed when it did not identify a risk that caused serious complications in the 131st patient in a phase 3 treatment trial after all had gone well with the first 130. To address the apparent disconnect between animal and human clinical studies, preclinical investigators must be more rigorous in developing their experimental designs and place more importance on identifying treatment complications.

### Dusty and Rusty

The GRMD model can be traced to a litter of golden retrievers, three males and one female, born July 21, 1981, outside Athens, Georgia, the home of the University of Georgia. Stiffness and simultaneous advancement of the pelvic limbs (bunny hopping) was noted in all three males at 9–11 weeks of age. Clinical signs of muscle disease were not seen in the dam, sire, or female littermate or in previous or subsequent litters of the dam. However, the same mating was purposely not repeated. A single male sibling from both the dam’s own litter and that of the grand dam had similar clinical signs. Cardinet and Holiday reported partial results from histopathologic studies of the male sibling from the dam’s litter in their 1979 monograph on canine muscle disease [82]. The sibling from the grand dam’s litter was euthanized after only a minimal evaluation and a necropsy was not done.

A veterinarian in private practice near Athens evaluated the dogs and submitted blood for routine analysis through the Veterinary Medical Diagnostic Laboratory at the University of Georgia on September 29, 1981. The most significant abnormality was dramatic elevation of serum CK (U/l) in two of the males, Dusty (16,770) and Rusty (24,442). Values for the other male (798) and female (529) were within normal limits. An inherited degenerative myopathy was suspected, and the owners were given a guarded prognosis. The male dog with the normal CK value died acutely when it was 10 weeks old, and a congenital diaphragmatic hernia compatible with an apparently unrelated syndrome seen in golden retrievers [83] was diagnosed at necropsy. Histopathologic lesions were not seen on assessment of selected skeletal muscles.

Dusty and Rusty were donated to the University of Georgia Veterinary Teaching Hospital on October 16, 1981, with an understanding that breeding might be done to perpetuate the condition. A series of tests, including CBC, serum chemistries, EMG, and muscle biopsies, were subsequently performed over the next 10 months. Results were compatible with a degenerative myopathy that had previously been documented in five other male golden retrievers (Table 1). The condition was called “golden retriever myopathy,” in keeping with a tendency for veterinarians to name canine diseases for the breed in which they occur [84]. Dusty and Rusty were moved to the College of Veterinary Medicine at North Carolina State University (NCSU) in the Fall of 1982 for continued assessment. Longitudinal phenotypic studies on these dogs up to 27 and 40 months of age at Georgia and NCSU were reported [85]. Briefly, serum CK was dramatically elevated (>10,000 U/l) and there were features of both muscle fiber degeneration (hyaline fibers, myophagocytosis) and regeneration (small basophilic fibers) on light microscopy. Persistent spontaneous high-frequency discharges, termed pseudomyotonia in our original paper, were seen on EMG.

**Table 1:** Additional golden retrievers with degenerative myopathy as of 1988

Age at clinical onset	Gender	Clinical signs	CK	EMG	Pathologic lesions	Outcome	Reference
10 days	M	Body stunting; glossal, shoulder, and neck muscle hypertrophy; bradycardia	NE	NE	Gross body stunting and muscle hypertrophy. Skeletal muscle histologic evidence of variation in fiber size, hyaline fibers with calcification, and basophilia consistent with regeneration; inflammation including giant cells; milder lesions in the heart.	Euthanasia at 10 days	[88]
6–8 weeks (two littermates were described)	M	Stiff gait with progression to a stilted, shuffling gait by 3 months. Fatigue with exercise, occasional respiratory distress, neck stiffness, resistance to jaw opening, and glossal hypertrophy.	Elevated	“Myotonia”	Severe degenerative muscle disease: myofiber necrosis, mononuclear phagocytosis, giant cells, and calcification	Euthanasia at 6–9 months when the dogs were still ambulatory	[91]
4–5 months	M	Generalized muscle atrophy, stiff gait, dysphagia, stenotic breathing, exercise intolerance.	NE	CRDs	Gross muscle atrophy; scattered necrotic fibers undergoing phagocytosis, increased endomysial connective tissue, calcification of myofibers, targetoid fibers, and fiber type grouping; myocardial degeneration and calcification	8 months at biopsy; outcome not given.	[82]
8 weeks	M	Stiff, shuffling gait; simultaneous pelvic limb advancement (bunny hopping); overextension at the carpus, overflexion at the tarsus, and abduction of the paws; stifle adduction; hypertrophy of proximal limb muscles; mild vertebral kyphosis; trismus; drooling.	13,435 IU/l	CRDs and positive sharp waves	4 months: fiber size variation, hyaline and necrotic fibers with myophagocytosis; centrally nucleated fibers; basophilic fibers consistent with regeneration. 8 months: progressive changes with mineralization and endoymysial and perimysial fibrosis; fibers with chains of central nuclei; fiber grouping.	Euthanasia at 8 months	[93]

**Fig. 3:**
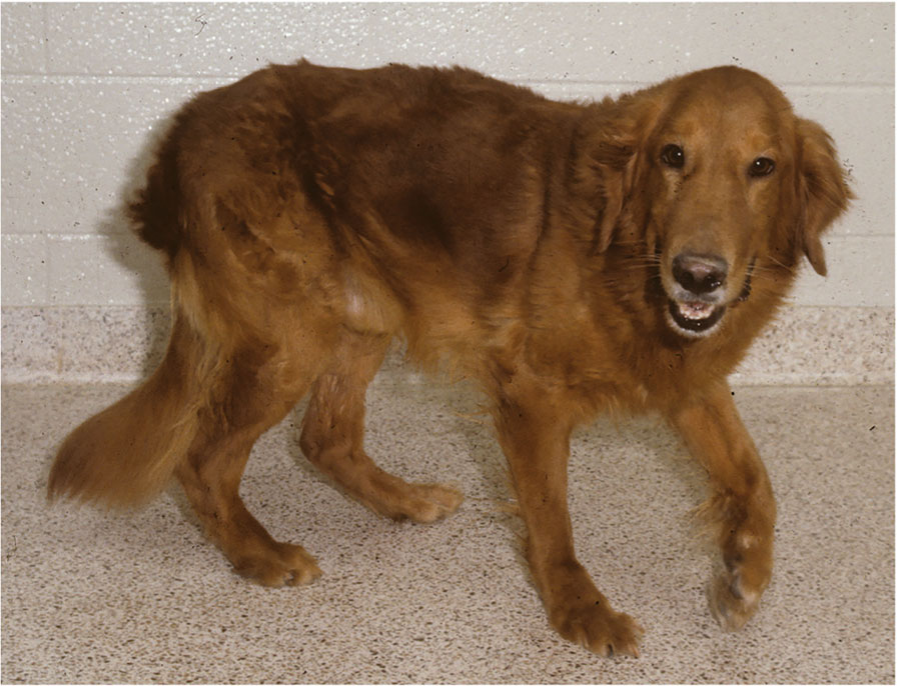
GRMD colony founder dog, Rusty, at North Carolina State University at ~3 years of age. Note the relatively mild phenotype characterized primarily by a plantigrade stance

Rusty (Fig. 3) was moved to the College of Veterinary Medicine at Cornell University at 41 months of age (see below). Clinical and pathologic data collected until his death at 6 years have been published [86, 87].

### Early reports of golden retrievers with apparent myopathy (Table 1)

Meier described a 10-day-old male golden retriever with an apparent myopathy in a monograph on canine muscle disease in 1958 [88]. The dog was born with an enlarged tongue that became bigger over time and “crowded the oral cavity and interfered with normal respiration and nursing.” Bradycardia and gross enlargement of the muscles of the neck and shoulders were identified on clinical examination. The most striking features at necropsy were body stunting and muscle hypertrophy. Variation in myofiber size, hyaline fibers with calcification, and myofiber basophilia consistent with regeneration were noted microscopically. Milder lesions were seen in the heart. There was a remarkable inflammatory cell infiltrate that included histiocytes and giant cells. Meier speculated that the findings were consistent with vitamin E deficiency at birth. Obviously, one cannot say definitively that this dog had a dystrophinopathy. While muscle hypertrophy, especially involving the tongue, is a feature of both DMD and GRMD, generalized muscle enlargement from birth is unprecedented in my experience. Otherwise, this dog bears some resemblance to the fulminant form of disease reported by Valentine et al. [86] and seen by others, in which respiration is compromised at birth. This condition may be analogous to pulmonary hypoplasia seen with congenital diaphragmatic hernia in humans [89]. Normal lung development is dependent on contractile activity of the diaphragm [90] so would likely be compromised in severely affected GRMD fetuses.

Alexander (Sandy) deLahunta provided clinical data on two male golden retrievers evaluated at Cornell University in his 1977 textbook, *Veterinary Neuroanatomy and Clinical Neurology* [91]. The syndrome was discussed together with a similar condition in Irish Terriers (see below) under a subheading of “Hereditary Myotonic Myopathy in Puppies.” Stiff gait was seen at 6 to 8 weeks of age, with progression to a stilted, shuffling gait by 3 months. Other clinical features included fatigue with exercise, occasional respiratory distress, neck stiffness, resistance to jaw opening, and enlargement of the base of the tongue. The dogs were still able to walk at 6 to 9 months when euthanasia was performed. Additional key findings included elevation of serum muscle enzymes, “myotonia” on EMG, and evidence of “severe degenerative muscle disease” on muscle biopsy or necropsy (necrosis of muscle cells, mononuclear phagocytosis, giant cells, and calcification). Reference was made to the more thoroughly studied condition in Irish Terriers, which had been shown by pedigree analysis to be X-linked [92] (see below).

**Fig. 4:**
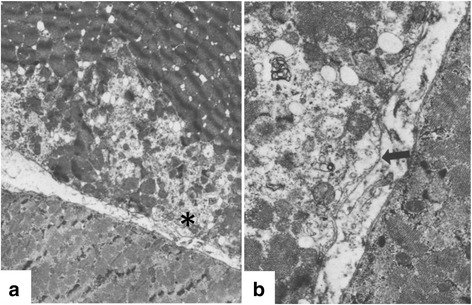
Myofiber with focal necrosis and membrane lesion in GRMD dog. Electron photomicrographs demonstrating a wedge-shaped subsarcolemmal area of disrupted architecture (**a**) and higher magnification (**b**) of the area identified with the *asterisk* where the myofiber membrane is absent (*arrow*) and the basal lamina is intact. Original magnification 4500 in **a** and 13,500 in **b**. Modified from reference [93]

Cardinet and Holliday described an additional 8-month-old golden retriever dog in 1979 [82]. Clinical features were consistent with those described by deLahunta. High-frequency discharges that waxed and waned were seen on EMG. Histopathologic features included scattered necrotic and/or calcified fibers. Cardinet and Holliday noted that fiber type grouping was consistent with a potential neuropathic component. Another dystrophic golden retriever with similar clinical signs was subsequently identified at Cornell and studied until 8 months of age [93]. Muscle ultrastructure was studied for the first time. Notably, focal wedge-shaped subsarcolemmal “delta” lesions and plasma membrane defects were illustrated (Fig. 4), in keeping with the membrane theory of DMD pathogenesis.

Thus, counting Rusty and Dusty, a total of seven golden retrievers with an apparent primary, inherited myopathy had been reported by the mid-1980s (Table 1). Additional dogs were cited via personal communication in our 1988 paper [85]. Strikingly, all affected golden retrievers were males, suggesting X-linked inheritance and potential genetic homology with DMD. Clinicopathologic evidence of serum CK elevation and small group muscle fiber necrosis and regeneration were also consistent with DMD. On the other hand, presence of CRDs on EMG, with associated stilted gait, stiffness, and marked muscle hypertrophy, were more consistent with myotonia.

### Cornell and canine X-linked muscular dystrophy

As detailed above, deLahunta and colleagues at Cornell played a key role in identifying early dystrophic golden retrievers. Barry Cooper, in particular, was motivated to establish a breeding colony of affected dogs and was aware of ongoing studies at NCSU. Rusty was sent to Cornell at 41 months of age, with an understanding that he would be used to establish a colony and then returned for follow on studies at NCSU. However, Rusty developed congestive heart failure at 5½ years of age and was euthanized at 6 years. So, instead, two obligate carriers (Dys and Trophy) and an affected male (Lewis) produced by Rusty were provided to NCSU to establish a second colony.

The studies by Dr. Cooper and his veterinary colleague, Beth Valentine, were instrumental in defining the phenotype of dystrophic dogs [86, 87, 94]. Rusty was initially bred with two female beagles and a larger retriever cross to produce mixed-breed obligate carriers. These carriers were then bred with Rusty or his male descendants to produce affected dogs. In this sense, GRMD is not a disease of pure-bred golden retrievers. We use the GRMD acronym to refer to the *DMD* gene splice site mutation present in affected dogs. Other acronyms, such as GSHPMD for German shorthaired pointer muscular dystrophy, refer to additional canine *DMD* gene mutations. Based on pedigree analysis of the dogs descendant from Rusty at Cornell, a likely recessive X-linked pattern of inheritance was established [95]. Phenotypic features in dogs from the Cornell colony were consistent with those reported in the previously described seven cases and included elevation of serum CK, CRDs on EMG, and histopathologic evidence of grouped muscle fiber necrosis and regeneration. Because this work was done before the *DMD* gene and dystrophin protein were identified, there was still a question as to whether the disease was a Duchenne genetic homologue. Genetic homology was established with reasonable certainly when neither *DMD* messenger ribonucleic acid (mRNA) nor dystrophin could be identified in dystrophic dogs [94]. Consistent with the GRMD site splice mutation later identified by Sharp et al. [96] (see below), endonuclease probes did not demonstrate RFLPS that would be expected with a large DNA deletion. Cooper and colleagues termed the disease canine X-linked muscular dystrophy (CXMD).

### Canine dystrophinopathies

Prior to the definition of the *DMD* gene and dystrophin protein, potentially analogous canine models were proposed based on an apparent pattern of recessive X-linked inheritance and consistent phenotypic features. In his 1977 textbook [91], deLahunta drew parallels between the condition in golden retrievers and an analogous disease in Irish terrier dogs reported by Wentink et al. [92]. The Irish terrier study included phenotypic data, extending from serum enzymes to necropsies, on a group of five male littermates. Pedigree data, suggesting a pattern of recessive X-linked inheritance, were reported from two prior litters from the same bitch. Serum enzymes, including aldolase and CK, were elevated, in keeping with the membrane theory of DMD pathogenesis. Wentink et al. specifically noted that the dramatic increase in CK was typical of DMD. Bizarre high-frequency discharges were seen on EMG, consistent with other canine dystrophinopathies that have since been characterized. Notably, dogs were variably affected, with two having a severe phenotype that necessitated euthanasia between 13 and 20 weeks of age, while another mildly affected dog lived until 16 months before euthanasia. Limb muscles were atrophied but the tongue was hypertrophied. One of the dogs had congenital peritoneopericardial diaphragmatic hernia at necropsy consistent with a syndrome later seen in GRMD dogs. Histopathological lesions were not seen in the hearts, central nervous system, or visceral organs of affected dogs. Based on extensive muscle histochemical and ultrastructural studies, Wentink et al. concluded that mitochondria were abnormal and speculated that uncoupled oxidative phosphorylation could be involved in disease pathogenesis.

**Fig. 5:**
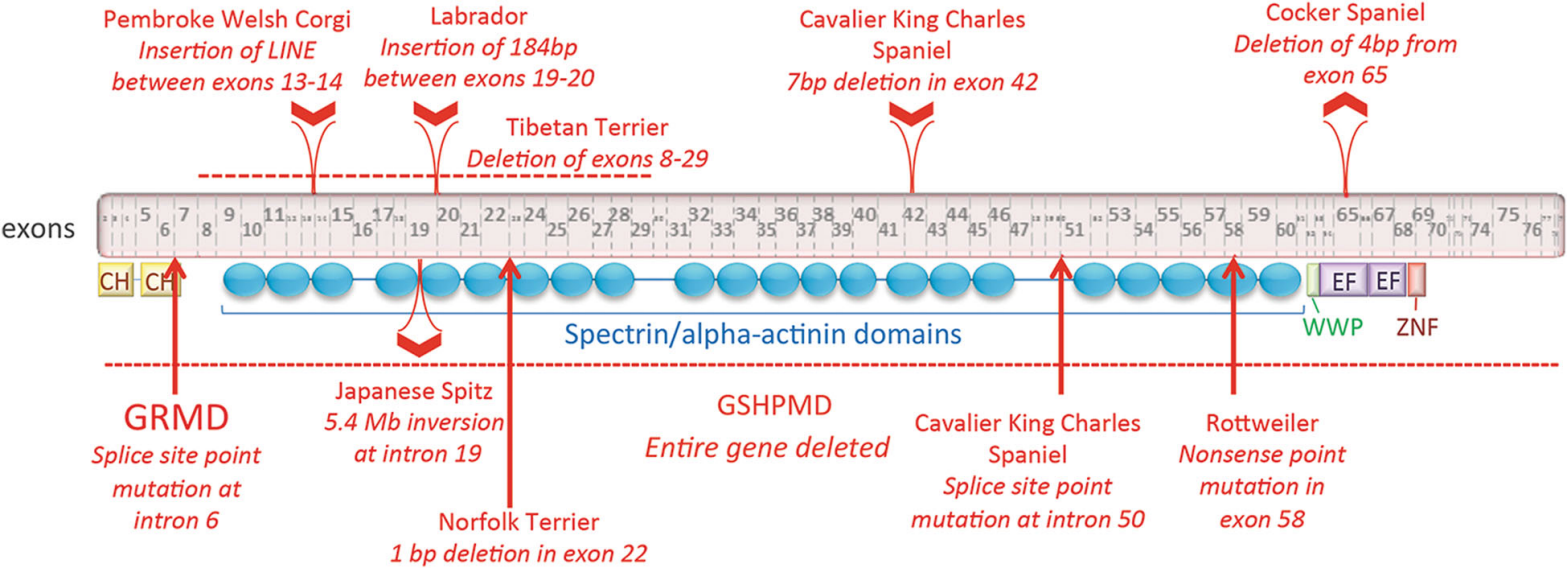
Canine dystrophin protein (Ensembl protein ID ENSCAFP00000031637), with mutation information for 10 dog breeds with dystrophinopathies. The breeds are Pembroke Welsh Corgi [98], Labrador retriever [97], Tibetan terrier [97], Cocker spaniel [97], golden retriever [96], Japanese Spitz [99], Norfolk terrier [100], German shorthaired pointer [101, 102], two distinct mutations in the Cavalier King Charles spaniel [103, 104], and Rottweiler [105]. *CH* indicates actin-binding calponin homology domains. The “WWP” domain binds proline-rich polypeptides and is the primary interaction site for dystrophin and dystroglycan. *EF* indicates members of the EF-hand family domain that stabilizes the dystrophin-dystroglycan complex. *ZNF* represents a putative zinc-binding domain, ZnF_ZZ is present in dystrophin-like proteins and may bind to calmodulin. All 79 exons are represented. Exons and protein domains are approximately shown to scale. Insertion and deletion mutations are shown above the exons. At the bottom of the figure, the German shorthaired pointer (GSHP) *DMD* gene deletion and point mutations are identified with a *hatched line and arrows*, respectively. Modified from reference [70]

Additional apparent dystrophinopathies have since been reported in a number of canine breeds [70, 96–105] (Fig. 5). Phenotypic findings, typical of those seen in GRMD, have included increased serum CK, CRDs on EMG, and histopathologic evidence of grouped muscle fiber necrosis and regeneration. When multiple dogs have been observed, disease severity has varied, in keeping with phenotypic variation seen in GRMD. As with GRMD, paradoxical muscle hypertrophy has seemed to play a role in the phenotype of affected dogs, with stiffness at gait, decreased joint range of motion, and trismus being common features. For the most part, dystrophinopathies in other canine breeds have not been defined beyond immunohistochemical and Western blot studies demonstrating the loss of dystrophin. Relatively few have been studied at the molecular level, with mutations largely paralleling the deletions, insertions, and splice-site mutations seen in DMD [70, 96–105].

The classification of canine dystrophinopathies is complicated by the tendency to associate DMD and BMD with severe and mild clinical phenotypes, respectively. All canine DMD deletions described to date have been out-of-frame, which would predict a severe DMD phenotype. As discussed above and further below, despite having an out-of-frame mutation, GRMD dogs may stabilize and live well into adulthood [75]. The GRMD phenotype could be influenced by spontaneous alternative splicing of the *DMD* transcript [106], with resultant production of truncated partially functional dystrophin isoforms expressed in so-called revertant fibers. Alternative splicing [107, 108] and revertant fibers [109] have also been reported in DMD. Conditions in which truncated isoforms of dystrophin are expressed have been reported in Japanese Spitz and Labrador retriever canine breeds. A 5.4-Mb fragment of the X chromosome is inverted at the level of intron 19 of the *DMD* gene and the *retinitis pigmentosa GTPase regulator (RTGP)* gene of Japanese Spitz dogs [99]. Transcription was demonstrated at the 5′ end but not beyond. A truncated 70–80 kDa protein, consistent with aberrant expression of Dp71 dystrophin-related protein, was identified. Baroncelli et al. described a 3-year-old male Labrador retriever with what was termed a Becker type syndrome [110]. The dog had a mild phenotype, normal serum CK, and widespread immunohistochemical evidence of dystrophin expression. Immunoblotting revealed two bands consistent with rod-domain and carboxyl-terminus fragments. The molecular mutation was not identified.

Similarly, in another report, Labrador retrievers with no dystrophin had increased serum CK and dystrophic lesions on biopsy but no clinical evidence of myopathy [111]. A somewhat analogous situation exists in German Shorthaired pointers that have a relatively mild phenotype even through the entire *DMD* gene is deleted [101, 102, 112]. Genetic mechanisms other than frame shift must underlie milder (or more severe) phenotypes in dystrophic dogs. A mild phenotype in two so-called *escaper* GRMD dogs from one of the Brazilian colonies was associated with a G>T mutation in the promoter region of the *Jagged1* gene [113]. Given that this substitution was apparently introduced by an outcross, the *Jagged1* mutation would not necessarily be found in other GRMD colonies. We routinely see mildly affected GRMD dogs that live beyond 3 years of age. In testing done to date, none of these dogs have had the *Jagged1* mutation. Importantly, the original founder dog for all GRMD dogs worldwide, Rusty, lived until 6 years when he developed congestive heart failure and was euthanized [86, 87]. Using Rusty as a benchmark, the GRMD phenotype actually appears to have worsened over time, potentially due to excessive inbreeding and concentration of the effects of genetic modifiers. The genetic basis of phenotypic variation is a subject of active research in my laboratory, with a recent genome wide association study (GWAS) identifying several potential candidate genes that could influence disease phenotype [114].

With the advent of molecular therapies, particular interest has been focused on certain so-called hot spot areas at exons 3-7 and 45-53 of the *DMD* gene (see above). In GRMD and the further outbred condition, canine X-linked muscular dystrophy in Japan (CXMD_J_) [115], an mRNA processing error results from a single base change in the 3′ consensus splice site of intron 6. Exon 7 is consequently skipped during mRNA processing [96]. The resulting transcript predicts that the dystrophin reading frame will be terminated within its N-terminal domain in exon 8. The dystrophin reading frame can be reestablished using antisense oligonucleotides that target exons 6 and 8 [42, 116]. With regard to the even more clinically relevant exon 45-53 hot spot area, Cavalier King Charles spaniels have a G-T missense mutation in the 5′ consensus splice site of intron 50 that results in deletion of exon 50 [103]. Antisense oligonucleotide-mediated skipping of exon 51 restored the reading frame and protein expression in cultured myoblasts from an affected dog. As mentioned above, the fascinating *DMD* gene deletional mutation in the German Shorthaired pointer breed bears particular mention. The entire *DMD* gene is deleted in affected dogs [101, 102], thus eliminating the possibility of alternative splicing and the confounding effects of revertant fibers.

### GRMD in preclinical trials: overview

Rather than repeat recent reviews of GRMD preclinical trials and biomarkers [77, 97], this discussion will focus on three areas: (a) the overall preclinical approach to DMD treatment development; (b) nuances of GRMD disease progression that must be considered in experimental design; and (c) cautionary lessons learned from prior GRMD treatment studies.

The DMD research community is fortunate to have two well-defined genetically homologous animal models that can be used in tandem to advance therapy development. Mdx mice should logically be employed for initial in vivo preclinical experiments. Studies that fail to demonstrate potential efficacy bode poorly for translation of the therapeutic strategy to humans and could provide a logical “stopping point” for continued development. Alternatively, if mdx findings are encouraging, consideration should be given to extending the work to GRMD or another canine model. However, in reality, studies in dogs are limited because of their availability and expense. When dystrophic dogs have been studied, results from mdx mice have not been consistently reproduced. In such cases, inherent species differences preclude saying whether the mouse or dog would better predict the ultimate outcome in humans. But, at the very least, a failure to reproduce favorable data in both species should raise concerns and prompt reassessment of the therapeutic approach.

The experimental design and outcome parameters used in preclinical trials should mirror those anticipated in human studies. Importantly, in the context of the “two cultures phenomenon” discussed above, animal studies should be rigorous, with close attention to issues such as group size, use of proper controls, and blinding. In a recent review of mdx and GRMD preclinical trials [77], we offered the following guidelines for improving translation of animal findings to humans:

- Design studies with sample sizes that are sufficiently powered to detect treatment effects with the outcome parameters used.
- Follow randomization and blinding procedures, including who is blinded and when.
- Provide details of the statistical methods used for data analysis and report all the results for each analysis.
- Develop reliable and sensitive primary and secondary endpoints for the animal model used.
- Independently validate treatment efficacy results in another laboratory.
- Validate treatment efficacy in two species (e.g., mdx mouse and GRMD dog for DMD) whenever possible.

### GRMD in preclinical trials: experimental design

**Fig. 6:**
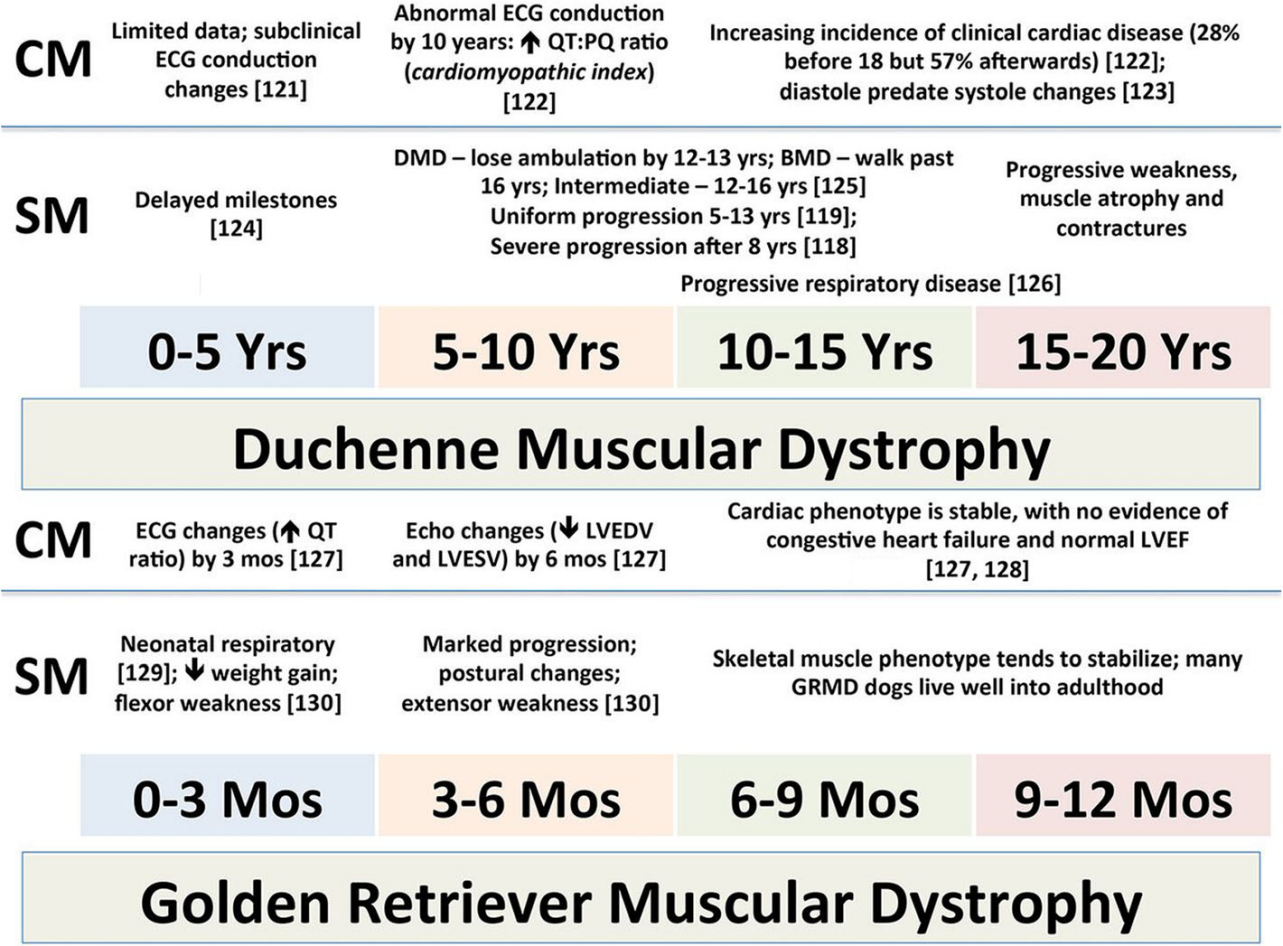
Comparative disease course of GRMD based on the relative equivalency of the first year of a golden retriever’s life and initial 20 years of a human’s. The two periods are divided into quartiles, e.g., 0–3 months of GRMD paralleling 0–5 years of DMD, with signs of the skeletal myopathy (SM) and cardiomyopathy (CM) listed for each period [118–130]. Note, the GRMD clinical course from 0–6 months largely parallels that of DMD over the 0–10 year period. However, the GRMD and DMD phenotypes then dramatically diverge, with GRMD dogs often stabilizing and DMD continuing to progress. *SM* skeletal myopathy, *CM* cardiomyopathy, *LVEDV* and *LVSDV* left ventricular end diastolic and systolic volumes, *LVEF* left ventricular ejection fraction. Modified from reference [75]

For the sake of GRMD trials, we have long focused on the 3- to 6-month age period during which clinical signs progress particularly rapidly. Based on an epidemiologic study by Patronek et al. [117], the first year of a golden retriever’s life roughly equates to 20 years of a human. Therefore, the GRMD age quartiles of 0–3, 3–6, 6–9, and 9–12 months would logically correspond to 0–5, 5–10, 10–15, and 15–20 years of DMD. In comparing the relative severity of disease over these age periods, GRMD and DMD generally progress analogously up to 6 months (GRMD) [75] and 10 years (DMD) [118, 119] (Fig. 6). While GRMD pups may be weak at birth and require dietary supplementation [120], they tend to stabilize around 2 weeks and are mildly affected up to 3 months, when more progressive clinical involvement is seen. Similarly, DMD boys have relatively stable disease up to 6 years of age and then progressively decline for the next 5 years [118].

In our experience, the GRMD and DMD phenotypes diverge after 6 months and 5 years, respectively, with dystrophic dogs tending to stabilize and boys inexorably progressing [75, 118–130]. Even though GRMD dogs in our colony progress markedly up to 6 months, they rarely lose the ability to walk, which would be analogous to becoming confined to a wheelchair in DMD, and often live well into adulthood. With this said, other laboratories have reported a more severe GRMD phenotype. For instance, ~25% of GRMD dogs produced in the colony at Alfort in France lose ambulation by 6 months [131]. In a similar vein, GRMD dogs in the Brazilian colony directed by Mayana Zatz generally die within the first 2 years of life [113]. Notably, both the French and Brazilian colonies were derived from the same founder dog, Rusty (see above), identified by our group in 1981. This distinction in phenotypic severity likely occurs due to the relative role of genetic modifiers in each colony, potentially being concentrated by inbreeding or introduced by outbreeding. As an example, inbreeding tends to worsen the phenotype of GRMD pups [120].

Planning and interpretation of (pre)clinical trials in both DMD and GRMD is confounded by variable disease progression among individuals. As with the differing phenotypes seen in GRMD colonies, DMD phenotypic variation likely occurs due to genetic modifiers. Polymorphisms in two genes, secreted phosphoprotein 1 (*SPP1*) (osteopontin) and latent transforming growth factor β binding protein 4 (*LTBP4*), have been shown to influence the DMD phenotype [132]. Per the discussion above, Vieira et al. identified the *Jagged1* gene as a modifier of the GRMD phenotype [113]. We recently published results of a GWAS that defined a number of genes associated with phenotype [114].

For local (intramuscular) or regional (single limb) therapeutic approaches, the opposite untreated limb can serve as a control. With systemic treatments, it is difficult to distinguish a treatment effect from inherently mild clinical disease. Speaking generally, the number of subjects required to establish benefit tracks directly with the standard deviation of the outcome parameter being used and inversely with the relative benefit anticipated. In a somewhat sobering review, Brooke et al. concluded that for a DMD clinical trial intended to “test a drug which might slow the disease to 25% of its original rate of progression, two groups (placebo and treatment) of 40 patients each would have to be followed for a year” [118]. Such a study typically determines the longitudinal effect of a treatment by comparing baseline and end-of-study values. This helps to remove the confounding effect of phenotypic variation because each individual essentially becomes his own control. In principle, even more cases would be required if only data from a final outcome parameter were compared between control and treatment groups.

Power analysis has been used on a limited basis to assess GRMD preclinical biomarkers, with only tibiotarsal joint (TTJ) tetanic torque and the 6-minute walk test (6MWT) being evaluated. The power analysis in our TTJ study anticipated that a single measurement would be done at the end of treatment and compared with a value from a control group [130]. Data for TTJ tetanic flexion torque showed that groups of 15 and 5 GRMD dogs would be necessary to demonstrate differences of 0.2 and 0.4 at 6 months, with associated powers of 0.824 and 0.856. Based largely on these natural history data, we have typically included 5 or 6 dogs in our GRMD preclinical trials. However, the natural relative recovery of TTJ flexion torque between 3 and 6 months complicates its use in GRMD preclinical trials [130]. Extension values decline over this same period but tend to vary more markedly among dogs, necessitating larger group sizes to establish significance. With more careful monitoring of inbreeding coefficient for sire-dam pairs in our colony, the overall GRMD phenotype is now less severe and not as variable as it was at the time the TTJ torque data were published [130]. A subsequent trial of prednisone in GRMD dogs demonstrated therapeutic benefit of ~60% (*p* < 0.05) for extension torque using groups of 6 treated and 10 untreated GRMD dogs [133].

For sake of the 6MWT, GRMD dogs walked significantly shorter distances than normal littermates at 6 and 12 months, generally supporting its use in preclinical trials [134]. We also determined that percent increase in CK after the 6MWT was higher in GRMD versus normal dogs, providing an additional potential biomarker. With this said, neither the 6MWT nor CK percent increase was particularly sensitive. Assuming a group size of 6 each for treated and control GRMD dogs, power analysis revealed that an 80% increase in mean height-adjusted distance walked or 55% decrease in mean post-exercise CK would be necessary to achieve a desired power of 80%.

As with longitudinal DMD trials, a GRMD study comparing values at baseline (3 months) and end of treatment (6 months) would lessen the effects of phenotypic variation, reducing the number of dogs required. Ideally, treatment groups would also be balanced between mildly and severely affected individuals, based on an early biomarker that tracks with disease. In a GRMD study focused on identification of early disease biomarkers, increased numbers of a subset of circulating T cells and decreased stride frequency on accelerometry at 2 months strongly associated with more severe clinical disease at 6 months [131]. While independent control groups for each preclinical trial should ideally be assessed, budgetary and animal number limitations may necessitate the use of natural history controls. In one such study, TTJ extensor torque and other indices were improved in six GRMD dogs treated with a nuclear factor kappa-light-chain-enhancer of activated B cells (NF-κB) inhibitor compared to an independent control group [135].

Just as the overall disease course for DMD patients and GRMD dogs varies, individual muscles also have different patterns of progression. Proximal muscles are classically affected in DMD, with further selective involvement of extensors versus flexors. Muscles, such as the quadriceps, that undergo eccentric contraction are particularly vulnerable [136]. Differential muscle involvement was well illustrated in our natural history TTJ torque study, with flexors being preferentially affected at 3 months and extensors at 6 months [130]. Pointing to the challenges one faces in analyzing functional data, the prednisone-treated GRMD dogs discussed above had a paradoxical decrease in flexor torque. This presumably occurred because of a reduction in early necrosis that otherwise would have led to functional flexor hypertrophy [133]. A similar decrease in flexor torque was seen in another study of GRMD dogs treated with prednisone and cyclosporine [137].

The pattern of differential involvement among skeletal muscles extends to the heart, which has a much later onset of clinical disease. Reasons for preferential disease of skeletal muscles or sparing of the heart are unclear but likely also involve different patterns of gene expression. Through a collaboration with investigators at Vanderbilt, we showed that brain-derived neurotropic factor (BDNF) is preferentially increased in young GRMD dogs, perhaps in keeping with a palliative cardiac effect. Moreover, BDNF levels tracked with increased ejection fraction in DMD patients [138]. The relatively late onset of cardiac dysfunction complicates preclinical GRMD studies done over the 3- to 6-month age period. Definition of cardiac biomarkers that would distinguish early subclinical disease is an active area of investigation for my laboratory.

Results of DMD [118] and GRMD [120, 139] functional tests track and correlate with each other, providing some assurance that they are valid markers of disease and can be used to document benefit. For sake of GRMD, mildly affected dogs have proportionally larger TTJ angles and tetanic extensor torque. In addition, cranial sartorius circumference measured at the time of surgical biopsy is proportionally smaller. The opposite pattern is seen in severely affected dogs. On the other hand, 6MWT results from a much smaller group of GRMD dogs did not correlate with other tests, causing concern about this test’s reliability [134].

### GRMD in preclinical trials: cautionary lessons

#### Pharmacologic approaches

In developing drug therapies for DMD, approaches have been based on somewhat intuitive assumptions that targeting secondary effects of dystrophin loss, like inflammation, myofiber necrosis, and muscle atrophy, would have benefit. While such treatments are logical, they may inherently compromise or augment ongoing homeostatic mechanisms and potentially *do more harm than good*. Studies in the GRMD dog have sometimes brought side effects into clearer focus, potentially allowing the particular treatment to be modified.

##### Anti-inflammatories

Glucocorticoids, such as prednisone, have become the standard of care for DMD [140, 141], but also cause a range of side effects [140, 142]. We were motivated to study the effects of prednisone in GRMD dogs to establish a baseline for comparing other treatments and to explore alternative dosage regimens that might not be practical in DMD. The study employed a variation of what has become our standard approach to preclinical studies, with dogs being dosed daily from 2 to 6 months of age at either 1 or 2 mg/kg [133]. While this dose is higher than the typical regimen of 0.75 mg/kg/day used in DMD [140, 141], care must be taken in extrapolating drug dosages among species. Body surface area takes into account variables such as drug metabolism and, therefore, offers a better guide than body weight in considering appropriate dosing [143]. Several functional and histopathologic biomarkers were compared between treated and control GRMD dogs at 6 months. Although a dose-related increase in TTJ extensor force was seen, there was a paradoxical decrease in flexor values. The reduced flexor force was attributed to a reduction in early necrosis and a less pronounced regenerative response that would otherwise have led to functional hypertrophy. Dogs in the 2 mg/kg group also had dramatic myofiber mineralization, potentially due to decreased clearing by macrophages. A subsequent GRMD study by another group of combined prednisone, again at 2 mg/kg, and cyclosporine produced similar results [137]. The fact that high dose prednisone has deleterious effects is not surprising and consistent with broader side effects of the drug. The differential effect on flexor and extensor function is more concerning because of the potential that this could translate to DMD patients. As discussed further below in section “Myostatin inhibition”, treatments that cause differential extensor and flexor muscle improvement or decline could aggravate preexisting contractures and postural instability.

Prednisone acts, in part, by inhibiting the NF-κB signaling pathway [144], which is activated in DMD [145]. Accordingly, this pathway has been targeted in preclinical DMD studies. We conducted a GRMD trial that built on prior mdx mouse experiments in which the Nemo Binding Domain (NBD) peptide was used to inhibit NF-κB signaling [135]. As with the prednisone study, treatment began at 2 months of age and extended for 4 months, with dogs being dosed intravenously three times weekly and studied at 6 months using several biomarkers. Results paralleled those seen with prednisone, in that TTJ extensor force was increased in treated versus control dogs but flexor values were decreased. Of greater concern, NBD administration over time led to infusion reactions in both treated GRMD and wild-type dogs, pointing to the potential for recombinant proteins to induce an immune response.

##### Inhibition of protein degradation

Given that DMD is predominantly a muscle-wasting disorder, degradative enzymes of the ubiquitin-proteasome (UPS) and calpain systems have been targeted therapeutically. Promising results have generally been achieved in the mdx mouse with inhibition of the UPS [146] but not the calpain system [147]. In a GRMD study, only about half of the muscles studied had increased calpain activity and most actually had decreased proteasome activity [148]. Of greater concern, numerous UPS components were decreased in the heart, suggesting that pharmacologic inhibition of these systems in DMD could have unintended deleterious consequences. Not surprisingly, GRMD dogs treated with a novel calpain inhibitor did not improve [149].

##### Myostatin inhibition

**Fig. 7:**
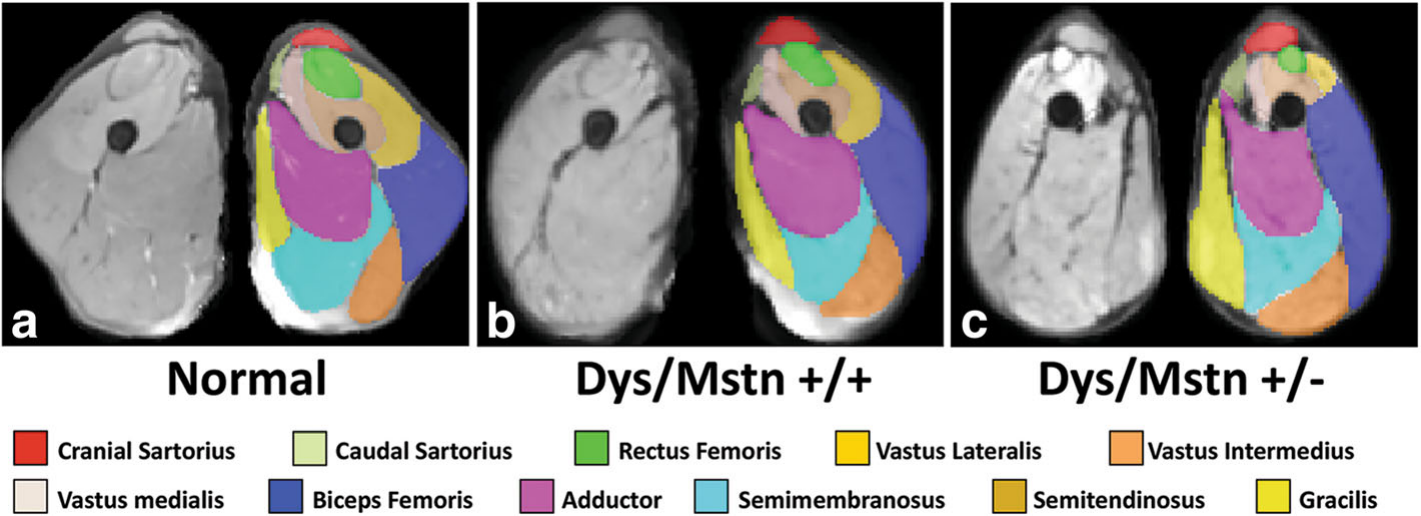
Averaged MRI segmentation of dogs with variable GRMD and myostatin (*Mstn*) genotypes. T2-FS MRI images of pelvic limb muscles in the transverse plane at the level of the midthigh are shown in non-dystrophic control (**a**), dystrophic GRMD, wild-type *Mstn* ^*+/+*^ (**b**) and GRMD, heterozygous null *Mstn* ^+/−^ (*GRippet*) (**c**) dogs. Note the proportional enlargement of the sartorius and hamstring muscles and the associated atrophy/hypoplasia of the quadriceps femoris of the GRMD, wild-type *Mstn* ^*+/+*^ dogs, relative to the non-dystrophic control dogs, and the even more dramatic differential size of these muscles in the *GRippet Mstn* ^+/−^ dogs. From reference [158]

Inhibition of the myostatin gene (growth and differentiation factor 8), a key negative regulator of muscle growth [150, 151], offers another approach to reverse muscle atrophy. Mdx mice in which myostatin was knocked out [152] or postnatally inhibited [153] had a less severe phenotype. Data from normal [154] and GRMD [155] dogs seemed to substantiate potential value of myostatin inhibition. However, likely reflecting feedback mechanisms, myostatin gene levels are already markedly lower in DMD patients [156], mdx mice [157], and GRMD dogs [139], raising questions about whether further inhibition is desirable. Such concerns were substantiated by a study in our colony in which GRMD dogs were bred with whippets carrying a myostatin gene mutation. We expected that myostatin heterozygous GRMD dogs (*GRippets*) would have an improved phenotype, consistent with the prior mdx work. Surprisingly, the *GRippets* had more severe postural changes than their dystrophic myostatin-wild-type littermates, apparently due to differential effects on agonist and antagonist muscles. For almost all muscles, the degree of atrophy or hypertrophy in GRMD dogs was more pronounced in the *GRippets* (Fig. 7) [158]. In keeping with studies showing that myostatin is already downregulated in GRMD, mRNA and protein levels did not differ between the two groups. For sake of interpreting these data, it is critical to recognize that findings from a study in which myostatin was downregulated in utero will not necessarily extrapolate to postnatal treatments. But, these findings emphasize that the dystrophic body is already motivated to downregulate myostatin and that the effects of inhibition on muscle mass will not necessarily be uniform.

#### Cell therapies

Therapies using muscle-derived or pluripotent stem cells provide a direct means to replace muscle. Because of their outbred nature, dogs are more likely than mice to model the immune response to cell-based therapies. Indeed, as discussed further below, the dog has proven superior to even primates in modeling host versus graft disease in bone marrow transplantation. Thus far, the most notable stem cell approach for DMD involved intramuscular transplantation of satellite cell-derived myoblasts from normal individuals into dystrophic muscles. Based on proof of principle studies in the mdx mouse [159], several human trials were instituted, with largely disappointing results [160, 161]. Most transplanted cells probably died due to the combined effects of poor blood supply and immune rejection; those that survived demonstrated limited migration [162–164]. We characterized canine myoblasts [165] and conducted analogous transplantation studies in the GRMD model during the 1990s. Presumably because of these same limitations, little implantation of donor cells was demonstrated. Failed translation from mice to humans (and dogs) may have related to variables influenced by scale, such as cell migration [78]. With this lack of success in GRMD, we developed a separate model system, in which muscles of normal dogs were injected with a mix of myoblasts and the toxin notexin. While myoblast implantation and differentiation was achieved, providing some hope for the overall approach, further studies have not been done [166].

Given the early disappointing results of myoblast transplantation and the inherent limitations of localized cell therapy to achieve meaningful benefit, considerable interest has focused on other stem cells that could potentially be delivered systemically. Bone marrow transplantation was a natural candidate, and initial findings were encouraging. In two separate studies from Gussoni et al., dystrophin expression and donor-derived myonuclei were demonstrated in transplanted mdx mice [167] and a DMD patient who received a bone marrow transplant for concomitant severe combined immune deficiency [168]. Nonetheless, in a follow-up study using the *mdx4cv* mutant that lacks revertant fibers, myofiber dystrophin expression never exceeded 1% over the 10-month post-transplant period [169].

As noted above, normal dogs have played a major role in the development of bone marrow transplantation as a reliable therapy, with many of the pioneering studies being done at the Fred Hutchinson Cancer Research Center in Seattle [170]. Thus, the Seattle group was particularly well situated to explore the utility of bone marrow transplantation in dogs with muscular dystrophy. To this end, GRMD/CXMD carriers from the University of Missouri and Cornell were used to establish a colony in Seattle. In a subsequent study, dystrophic dogs with allogenic bone marrow engraftment did not have increased dystrophin-positive fibers, wild-type dystrophin RNA, or donor-derived myonuclei [171].

Although results of bone marrow transplantation have thus far been disappointing, other stem cell therapies have been more encouraging. The GRMD model has been increasingly utilized in these studies, with generally positive findings being reported in 2016, alone, for mesoangioblast [172], adipose mesenchymal [173], and muStem [174] cells. Despite these promising results, analogous studies have not yet been translated to DMD patients [175]. Much as with bone marrow transplantation, the dog’s outbred nature has been ideal for defining potential immunologic adverse effects. Considering that myoblasts may elicit an immune response due to both major histocompatibility complex (MHC) and non-MHC antigens [176], most stem cell studies in dystrophic dogs have employed immunosuppressive regimens. Findings from these studies have been confounded by the potential for anti-inflammatories to have an independent beneficial effect. This conundrum arose in a 2006 study in which GRMD dogs treated systemically with mesodermal stem cells derived from the vascular wall, so called mesoangioblasts, had dystrophin expression [177]. Functional improvement in these dogs was questioned, given that the benefit could have occurred due to immunosuppression [178]. Indeed, in a follow-up report, GRMD dogs treated with cyclosporine and prednisone alone had analogous improvement [137]. Another study of MuStem cells contrasted functional outcome variables in immunosuppressed and control dogs and found that dogs treated with stem cells had greater therapeutic benefit [179].

Due to the confounding effect of anti-inflammatory drugs, some subsequent stem cell studies in dystrophic dogs have foregone immunosuppression, choosing instead to match donor-recipient pairs for the canine MHC, the dog leukocyte antigen [173, 180, 181]. Cell implantation and at least minimal dystrophin expression have been achieved in these dogs, with minimal or no adverse immunologic response. Another recent study offers an additional cautionary note [182]. Non-immunosuppressed GRMD dogs were treated three times intra-arterially with autologous CD133+ cells engineered with a lentivirus cassette to skip exons 6-8 and, thereby, restore the mRNA reading frame. Timed function biomarkers stabilized or improved in treated versus control GRMD dogs until the third injection, after which a precipitous decline was seen. This deterioration was attributed to an adaptive immune response aggravated by dystrophin acting as a neoantigen, analogous to a reaction seen in a DMD adeno-associated virus (AAV)-micro-dystrophin gene therapy trial (see below).

#### Genetic therapies

Genetic therapies have included AAV-mediated insertion of a truncated mini/micro-dystrophin to fit the vector’s limited ~4.5 kb carrying capacity, antisense oligonucleotides to induce exon skipping and reestablish the dystrophin reading frame, agents to read-through stop codon mutations, and replacement of dystrophin at the sarcolemma with surrogates such as utrophin. Studies in the mdx mouse have generally demonstrated efficacy and safety of these strategies. Some therapies have subsequently been extended to the GRMD model, providing further proof of concept [77, 97] (see discussion under canine dystrophinopathies above). Data from dystrophic dogs supported the antisense approach, and side effects were not seen [116, 183–186]. But, even with supportive mdx and GRMD preclinical data, human trials have thus far been inconclusive [59, 60].

Therapies employing AAV-mini/micro-dystrophin constructs should, in principle, offer similar therapeutic benefits and potential side effects as those employing exon-skipping strategies. With that said, while supportive data have again been demonstrated, greater safety concerns have been identified. Initial alarm was caused when localized (intramuscular) treatment elicited an immune response to either AAV capsid antigen [187] or dystrophin serving as a neoantigen [188] that could be blocked with immunosuppression [189]. Providing encouragement, dystrophin expression was demonstrated after intramuscular injection of an AAV-micro-dystrophin construct without immunosuppression in a single dystrophic dog [190]. And, in another study, immunosuppressed dystrophic dogs treated by intramuscular injection with AAV-micro-dystrophin had improved force and lower eccentric contraction decrement (ECD) despite a nonspecific lymphocytic infiltrate [191].

Interestingly, the immune response has generally been less pronounced with subsequent regional limb and systemic therapies, potentially because of a dilutional effect on the causative antigen [97]. However, one GRMD dog treated with AAV-mini/micro-dystrophin by regional limb delivery by my group in collaboration with Xiao Xiao at the University of North Carolina-Chapel Hill had increased signal intensity on MRI compatible with edema 3 months after treatment, and the level of dystrophin expression was actually higher in the contralateral limb (Kornegay JN and Xiao X, unpublished). This likely reflected the combined effects of dampened dystrophin expression due to the immune response and leakage at the tourniquet to allow systemic delivery. Given the generally promising results of regional limb delivery in GRMD dogs, phase 1-safety studies using saline versus active construct have been completed in both the lower [192] and upper [193] extremities of adult muscular dystrophy patients, with minimal side effects.

**Fig. 8:**
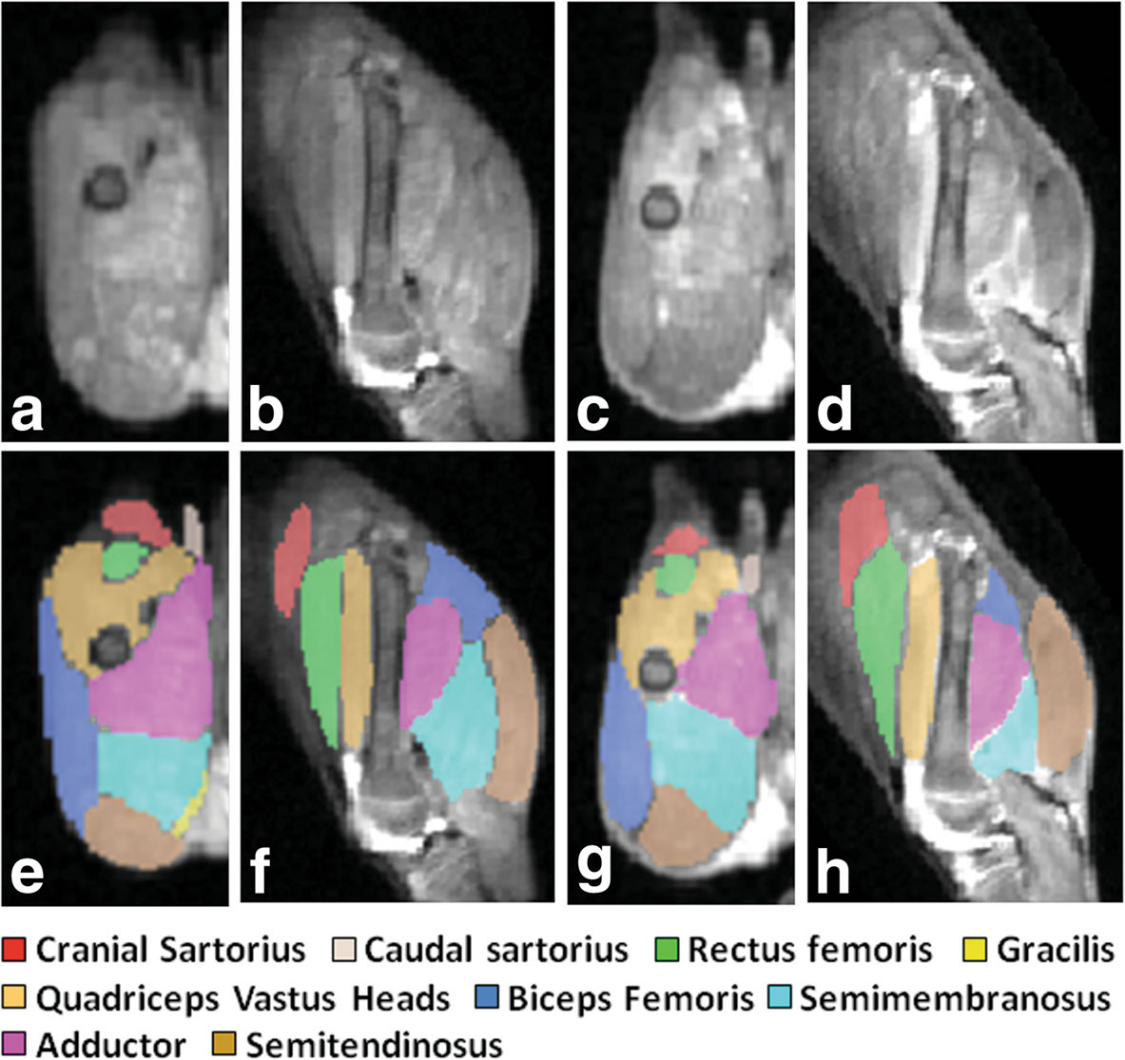
T2-weighted MR images of GRMD pelvic limb muscles 16 weeks after AAV9-CMV-mini-dystrophin vector intravenous injection. Transverse (*left*) and sagittal (*right*) images of two different dogs (dog 1: **a**, **b**, **e**, and **f**; dog 2: **c**, **d**, **g**, and **h**) are seen. The images in **e**–**h** have been segmented and color-coded to outline individual muscles. Signal intense lesions that persisted with fat saturation, most likely representing fluid due to inflammation or edema, are particularly pronounced in the vastus heads of the quadriceps and adductor muscles. From reference [195]

Results of systemic AAV-mini/micro-dystrophin therapy in dystrophic dogs have been mixed. On the one hand, our group and others have demonstrated long-term dystrophin expression, with [194] and without [195] immunosuppression. But, one group of dogs that we studied had delayed growth and pelvic limb muscle atrophy and contractures, seemingly due to an innate immune response (Fig. 8) [195]. Moreover, clear evidence of functional improvement akin to that seen in GRMD dogs with the exon-skipping strategies [186] has not been demonstrated after systemic therapy with AAV-mini/micro-dystrophins, giving rise to persistent questions about the ability of mini/micro-dystrophins to sustain myofiber integrity when stressed by larger mammals.

## Conclusions

Duchenne muscular dystrophy (DMD) is a devastating X-linked disease for which ameliorative treatments are desperately needed. As with other inherited conditions, genetically homologous animal models are critical for exploring DMD disease pathogenesis and the efficacy and side effects of potential therapies. Until the *DMD* gene and dystrophin protein were identified in the 1980s, putative animal models were chosen based on similarities in the pattern of inheritance and common phenotypic features. Golden retriever dogs with an apparent X-linked degenerative myopathy, subsequently termed golden retriever muscular dystrophy (GRMD), were recognized in the 1970s and 1980s and shown to have phenotypic features consistent with those of DMD. These features, including elevated serum enzymes, sarcolemmal defects, and CRDs on EMG, were in keeping with the membrane theory of DMD disease pathogenesis. One of these dogs, Rusty, identified at the University of Georgia in 1981, was the founder for initial colonies at Cornell University and NCSU. Subsequent studies confirmed genetic homology with DMD, and multiple additional colonies were established in the USA and around the world. In a similar time frame, an additional naturally occurring DMD genetic homologue, the mdx mouse, was identified and characterized. Extending from these studies in the 1980s, the DMD research community has had these two well-defined genetically homologous animal models to use in tandem for therapy development. However, neither of these models is completely analogous, with the mdx mouse displaying a mild phenotype and the more severely affected GRMD dog often stabilizing after 6 months of age. Advantages of the mdx mouse relate to its consistent phenotype and relatively modest expense, allowing multiple variables to be tested through reasonably powered studies. On the other hand, the mouse’s small size may limit assessment of scalable variables such as cell migration or drug diffusion. Perhaps even more importantly, preclinical studies in mdx mice have generally not identified complications, most notably immunologic side effects of gene and cell therapies. These advantages and limitations are essentially reversed for dystrophic dogs. The expense of animal housing and required facilities limits the number of dogs that can be studied. Accordingly, multiple variables, as with the use of immunosuppression, cannot always be tested. Phenotypic variation further reduces the power that can be achieved with GRMD trials. On the other hand, the dog’s larger size and outbred nature allows for better modeling of scalable variables such as cell diffusion and the immune response to biologics. A general paradigm has evolved, whereby initial testing is done in mdx mice and, assuming positive results, follow-up studies are completed in dystrophic dogs. As with preclinical studies more generally, findings from these animal models have not consistently translated to DMD patients. Examples of failed translation include pharmacologic approaches, the myoblast transplantation studies of the 1990s, and the current uncertainty surrounding exon-skipping strategies. A major factor in this lack of translation has been termed the “two cultures phenomenon,” whereby experimental design in animal studies has not consistently mirrored the rigor of human clinical trials, with fundamental tenets such as appropriate powering of outcome parameters and blinding not being sufficiently considered. To address this ongoing problem, certain basic standards should be adopted. Care should be taken in planning the experimental design to ensure that sample sizes are sufficiently powered to detect treatment effects with the outcome parameters used; animals should be randomly assigned to treatment and control groups, and investigators should be blinded to which animals are treated; statistical methods used in data analysis should be carefully considered and reported; endpoints (biomarkers) should largely follow those to be used in future DMD trials and be studied in a natural history setting to allow appropriate powering in advance of the preclinical trial; results should be validated in another laboratory; and, especially for treatments such as cell and gene therapies that carry substantial risk, treatment efficacy and complications should be determined in both the mdx mouse and GRMD dog. In this last instance, the role of animal models in predicting side effects has often been overlooked or deemphasized. Following on the premise that physicians should first and foremost “do no harm,” preclinical studies offer an opportunity to identify potential risks that can be corrected prior to human trials. The GRMD model has played a particularly important role in characterizing complications of pharmacologic intervention and immunologic reactions to cell and gene therapies.

## Abbreviations

6MWT: 6-minute walk test
AAV: Adeno-associated virus
BMD: Becker muscular dystrophy
CK: Creatine kinase
CPK: Creatine phosphokinase
CRDs: Complex repetitive discharges
CXMD: Canine X-linked muscular dystrophy
CXMD_J_: Canine X-linked muscular dystrophy in Japan
DMD: Duchenne muscular dystrophy
EMG: Electromyography
FDA: Food and Drug Administration
*GRippets*: Cross bred myostatin-heterozygote and GRMD dogs
GRMD: Golden retriever muscular dystrophy
GWAS: Genome wide association study
IND: Investigational new drug
*LTBP4*: Latent transforming growth factor β binding protein 4
MHC: Major histocompatibility complex
mRNA: Messenger ribonucleic acid
NBD: Nemo Binding Domain peptide
NF-κB: Nuclear factor kappa-light-chain-enhancer of activated B cells
RFLPs: Restriction fragment length polymorphisms
*SPPI*: Phosphoprotein 1 (osteopontin)
TTJ: Tibiotarsal joint
UPS: Ubiquitin-proteasome system
